# Adolescents’ Strategies to Manage Mental Health Challenges In and After the Pandemic: Two Mixed-Methods Studies

**DOI:** 10.1007/s10802-026-01471-w

**Published:** 2026-06-09

**Authors:** Hilde M. Huizenga, Floor E. Ammerlaan, Indi Zandstra, Lynn Mobach, Helle Larsen

**Affiliations:** Department of Psychology, University of Amsterdam, Nieuwe Achtergracht 129 B, Amsterdam, 1018WS The Netherlands

**Keywords:** Adolescence, Protective factors, Managing mental health challenges, Mixed methods, Pandemic

## Abstract

Identifying the strategies adolescents use to manage mental health challenges can offer important insights into factors protecting against more severe difficulties. Yet little is known about adolescent-specific strategies to manage these challenges. To address this gap, we created an innovative online tool in which adolescents could share how they manage specific mental health challenges, that is, symptoms of hyperactivity/inattention, conduct problems, and emotional problems, and in which they could rate perceived helpfulness of their strategies. The first study, during the pandemic, investigated a community sample of 218 adolescents aged 16 to 19. Inductive analysis uncovered 51 strategies, some mirroring established emotion regulation or coping strategies, while others were novel. Strategies varied across symptoms; quantitative analyses indicated that the most frequently used strategies were considered helpful. To check for robustness, we performed a second study after the pandemic in an independent community sample of 118 adolescents aged 16–20. This study largely replicated the first study findings, suggesting that strategies used and rated as helpful during the pandemic were also used and rated helpful after it. In addition, the second study investigated whether perceived helpfulness was related to the specific goal of a strategy: problem solving or cognitive or experiential avoidance, which proved not to be the case. These two studies thus shed light on the wide array of adolescent-specific and symptom-specific strategies that adolescents find helpful. They offer valuable insights for designing prevention programs that may protect against more severe difficulties, as they resonate with adolescents’ unique lives and needs.

**Supplementary Information:**

The online version contains supplementary material available at 10.1007/s10802-026-01471-w.

## Introduction

Psychopathology often emerges during adolescence (Solmi et al., 2022). The resulting world-wide prevalence is high; about one in seven 10-19-year-olds experience psychopathology (Polanczyk et al., 2015; World Health Organization, 2021).^1^ The associated burden is considerable^2^. Consequently, psychopathology in young people is the main worldwide contributor to years lost to disability, far exceeding contributions of accidents and infectious and parasitic diseases combined (Gore et al., 2011). Moreover, the length of adolescent psychopathology episodes is predictive of recurrent episodes later in life (Patton et al., 2014). For these reasons, it is imperative to study factors protecting against psychopathology in adolescence.

An important protective factor is the set of strategies adolescents use to manage mental health challenges. That is, it has been suggested that adequate management of initial symptoms protects adolescents against development of more severe symptoms, and thus, diagnosed conditions (Aldao et al. 2010a; Belcher et al. 2021; Cavicchioli et al. 2023; Cole et al. 2014; Jefferies et al. 2023; Johnson 2012; Nolen-Hoeksema 1991; Rommelse et al. 2016). When studying these protective strategies, it is necessary to take into account three aspects. First, it is of importance to investigate protective strategies not only among adolescents diagnosed with psychopathology, but specifically among those without such diagnoses, as the strategies employed by non-diagnosed adolescents may be more protective compared to those utilized by diagnosed adolescents (Aldao et al. 2010a). Second, it is important to study strategies reported by adolescents themselves, as these may differ from those reported, or theorized, by adults (Shanahan et al., 2023). Third, it is valuable to study symptom-specific strategies, as strategies are likely to differ between symptoms. For example, adolescents may use different strategies to manage worrying (a symptom of emotional problems) than to manage lack of focus (a symptom of hyperactivity/inattention). Taken together, information is needed on adolescent- and symptom-specific strategies, particularly in a community sample.

Valuable information on strategies employed by adolescents can be obtained from qualitative studies, implementing open-ended questions, interviews or focus groups. A review aggregating 48 qualitative studies in adolescents and young adults diagnosed with psychopathology suggested general, thus not symptom-specific, strategies like making art, talking or writing about experiences, cognitive restructuring, doing enjoyable activities like listening to music or watching television, believing in yourself, and alcohol (mis)use (Woodgate et al., 2017), see also (Aass et al., 2021; Özkul & Günüşen, 2021; Ringer, 2019). Qualitative studies in adolescents in a community sample are however scarce: one recent qualitative study investigated strategies to regulate emotions in adolescents (Chang et al., 2023), but symptom-specific strategies have not been investigated.

Quantitative studies on established emotion regulation and coping strategies did investigate strategies used by adolescents with and without a diagnosed condition (Compas et al., 2017; Johnson, 2012; Schäfer et al., 2017). A meta-analysis summarized these strategies as: problem solving, emotional expression, emotional suppression, cognitive reappraisal, distraction, acceptance, avoidance, denial, wishful thinking, emotional modulation, unregulated release of emotions, and humor (Compas et al., 2017). Importantly, what these quantitative studies do not reveal, is detailed insight into the adolescent-specific nature of these emotion-regulation and coping strategies. As a hypothetical example, the established emotion regulation strategy “distraction” may consist of a variety of adolescent-specific strategies, for example watching Netflix and running. In addition to that, emotion regulation and coping may be one way to manage psychopathological symptoms, but other symptom-specific strategies may also exist. Again, as a hypothetical example, adolescents may adopt the strategy *avoid screentime* to manage lack of focus (symptom of hyperactivity/inattention), a strategy which may not be easily characterized as an existing emotion regulation or coping strategy.

Qualitative studies thus provided information on strategies predominantly used by adolescents with a diagnosed condition, whereas quantitative studies yielded information on established strategies used by adolescents with and without a diagnosis. What is lacking however is information on adolescent- and symptom-specific strategies, particularly those used by adolescents without a diagnosed condition.

To fill this gap, we aimed to elicit adolescent- and symptom-specific strategies in a large adolescent community sample. We used two criteria to determine the symptoms for which we would probe strategies. First, the list of symptoms should cover a wide range, that is, both internalizing and externalizing symptoms. Second, the list of symptoms should be short to enhance feasibility. We therefore selected symptoms from the Strengths and Difficulties Questionnaire (SDQ) (Goodman et al., 1998). The SDQ contains 15 symptom items probing difficulties in hyperactivity/inattention, conduct problems and emotional problems; it is used as a screener for symptoms related to the diagnoses of ADHD, conduct disorder, and anxiety/depression (Goodman et al., 2003; Muris et al., 2003; Theunissen et al., 2019). To assess how adolescents manage symptoms of each of these three difficulties, we reformulated SDQ items, see (Weems et al., 2021) for a similar approach in which they adapted an existing questionnaire to elicit open ended responses. For example, we reformulated the hyperactivity/inattention item “I am restless, I cannot stay still for long’’ into the open-ended question “If I am restless or if I cannot stay still for long, I use these fixes……”, after which adolescents had the opportunity to list strategies and to indicate how helpful they perceived these strategies to be. We implemented the resulting Symptom-specific Fixes Instrument (SFI) in an online format, which offered the possibility to gather data in a large sample. We adopted a mixed-methods approach to the analysis of the resulting SFI data (Plano Clark, 2017). Specifically, we analyzed the answers to the open-ended SFI questions qualitatively using inductive coding (Braun & Clarke, 2006), and we analyzed their helpfulness quantitatively.

The overarching aim was thus to investigate adolescent-specific strategies for managing symptoms of hyperactivity/inattention, conduct problems and emotional difficulties. More specifically, the first aim was to generate an inventory of such strategies in a large adolescent community sample. Second, we examined the extent to which these strategies align with established emotion regulation or coping strategy frameworks (Compas et al., 2017). In this manner we could identify detailed adolescent and symptom-specific instances of established strategies and could find additional adolescent- and symptom-specific strategies beyond these established strategies. Third, we examined whether the use of strategies varied depending on the specific symptom domain - hyperactivity/inattention, conduct problems and emotional difficulties (Compas et al., 2017; Zeman et al., 2002). Finally, we investigated whether adolescents considered their own strategies helpful. This self-reported helpfulness may provide a first indication on protectiveness of adolescent-specific strategies. We investigated these questions in two studies. Because we conducted Study 1 during the Covid-19 pandemic, we designed Study 2 to replicate this study in an independent post-pandemic sample, thereby allowing for a robust examination of these strategies.

By adressing these questions, this study aims to contribute valuable insights into how adolescent-reported strategies can act as protective factors against more severe mental health difficulties. Hereby, this research not only enhances our theoretical understanding of these protective factors in adolescence but also holds practical implications for prevention and intervention strategies, ultimately informing how adolescents can be supported to use their own strategies, or develop new ones, to manage their mental health difficulties.

## Study 1

### Methods

#### Materials

*Strengths and Difficulties Questionnaire (SDQ).* We characterized the sample by psychopathology symptoms as assessed by the Dutch translation of the self-report Strengths and Difficulties Questionnaire (SDQ) (Goodman et al., 1998; Van Widenfelt et al., 2003). The SDQ evaluates difficulties related to four subscales: hyperactivity/inattention, conduct problems, emotional symptoms, and peer problems. A fifth scale measures prosocial behavior (strengths). The questionnaire consists of 25 items where each scale is composed of five items. For example, ‘I am restless, I cannot stay still for long’ for the hyperactivity/inattention scale. Participants were asked to think back how things have been for them over the last six months and report for each statement whether this statement has been 0 - *not true*, 1 - *somewhat true*, or 2 - *certainly true* for them. Difficulty subscale scores thus ranged from 0 to 10. Higher scores implied greater difficulty.

The first three scales of the SDQ have been used as a screener for symptoms related to the DSM diagnoses ADHD, conduct disorder, and anxiety/depression (Goodman et al., 2003; Muris et al., 2003; Theunissen et al., 2019). Therefore, although the entire SDQ was administered, only these three subscales were analyzed. Cronbach’s alpha was acceptable for the subscales hyperactivity/inattention (0.75) and emotional symptoms (0.80), whereas it was low for the subscale conduct problems (0.28).

*Symptom-specific Fixes Instrument (SFI) .* To examine the strategies used by adolescents, the 15 SDQ items relating to the hyperactivity/inattention, conduct problems and emotional symptoms subscales were reformulated in open ‘If I…’ statements. For example, ‘If I am restless, I cannot stay still for long, I use these fixes……’. Participants had the opportunity to finish each statement by writing down a maximum of five strategies used over the last six months, no minimum required. They were also asked to rate how often these strategies were helpful for them, they did so on a scale of 1 – *rarely*, 2 – *sometimes*, 3 – *regularly*, and 4 – *often*. The SDQ item ‘I fight a lot. I can make other people do what I want’ was separated into two statements: ‘If I want to fight’ and ‘If I want other people to do what I want’. We did so because a pilot study revealed that participants were likely to answer strategies corresponding to only one of the two fragments composing the original SDQ item. This resulted in a total of 16 statements. If participants did not enter a single strategy on a statement, a message popped up encouraging them to reconsider and write down a strategy. However, the message also presented the option to write down ‘I don’t know’ if participants were unable to recall strategies. The SFI thus elicits open responses, whereas traditional coping or emotion-regulation measures require responses on a Likert scale. Therefore, psychometric analyses to assess reliability or factor structure are not warranted for the SFI.

#### Procedure

Participants were informed about the study through an information letter and an online informed consent form. They had to sign this form prior to starting the survey. In the SDQ and in the SFI we asked participants to think back over the previous six months. As this period from mid-July 2021 to mid-February 2022 featured increasingly restrictive COVID-19 limitations (RIVM, 2022), we briefly describe them in the Supplementary Online Materials (SOM). Data was collected online via a survey in Qualtrics (Qualtrics, Provo. UT). In the survey, the questionnaires were administered in the following order: first the SDQ, followed by the SFI, thereafter two unrelated questionnaires, and finally a questionnaire asking for demographics. On average it required 20 min to complete the whole survey. Due to the lockdown, teachers posted the survey on the online platform used to communicate with their students. Therefore, all participants filled in the survey outside the classroom without supervision by a teacher or researcher. To guarantee anonymity, we were not allowed to collect IP addresses.

## Data Analysis

### Characterization of the Sample

We checked whether the SDQ subscale scores on hyperactivity/inattention, conduct problems and emotional symptoms mimicked those in a sample gathered before the outbreak of the COVID-19 pandemic. We did so by comparing them to norm tables based on pre-pandemic data (Vugteveen et al., 2022). As these norm tables are gender and age (12–17 years) specific, respondents 18 and 19 years of age were compared to the norms of 17-year-olds. The norms provide a distinction between “normal” (0%-80%), “borderline” (80%-90%), and “abnormal” (90%-100%). Therefore, if the current scores match those in the normative sample, 80% of the participants are expected to belong to the first category, 10% to the second, and 10% to the third. We tested differences between observed and expected percentages with a binomial test for proportions.

### Coding of Strategies

The responses on SFI were first analyzed using a bottom-up inductive approach (Braun & Clarke, 2006). Coding was done by three Dutch female psychology students aged 21 to 23 years. To minimize disparities and disagreements, a calibration session was held using the data from a pilot survey, and data of 45 participants was double coded, followed up by a discussion. That is, each coder wrote in a logbook which responses were difficult to code. For example, for the ruminating item, one of the responses was “continue ruminating”. The logbooks list it could either be coded as “think or reason” or as “continue the undesired behavior”. Ater discussion, it was decided to use the latter code. For the same item, the response “throw a dice” was deemed to require too much interpretation and thus was treated as a missing response. The other data was divided over the three coders where each coder coded responses corresponding to one of the three SFI subscales relating to fixes for ADHD, conduct disorder, and anxiety/depression symptoms.

In this inductive approach, responses were grouped in strategies, this was done per symptom. For example, the responses “playing soccer” and “weight training” were grouped under the strategy *workout*. It was possible for responses to be grouped under more than one strategy. For example, the response “I will go for a walk or workout” was labelled *going for a stroll* and was also labelled *workout*. It was also possible for similar responses to be grouped under different strategies depending on the symptoms the response related to. For example, the response “putting away my phone” was labelled *eliminate distractions* when answered to the hyperactivity/inattention symptoms and was labelled *avoid screentime* when answered to the emotional symptoms. The helpfulness of a strategy was defined as the average helpfulness of responses belonging to that strategy. This resulted in a list of strategies per symptom, how many adolescents used each strategy and how helpful they rated those strategies.

Next, we used a top-down deductive approach to determine whether the strategies could be classified as the 12 established strategies identified in a meta-analysis on emotion regulation and coping strategies used by youngsters (Compas et al., 2017): problem solving, emotional expression, emotional suppression, cognitive reappraisal, distraction, acceptance, avoidance, denial, wishful thinking, emotional modulation, unregulated release of emotions, and humor.

### Participants

We studied a community sample of adolescents aged 16 to 19 years. Ethics approval was obtained before the study began (Faculty Ethics Review Board of the University of Amsterdam; project number 2021-DP-13023). This ethics committee required informed consent from participants but not from their parents. In exchange for completing the survey, participants were able to enter a lottery including four 20-euro gift cards. To guarantee anonymity, participants who wished to enter the lottery were redirected to a separate survey where they could enter their email address. University students that completed the survey were given a choice between entering the lottery or receiving school credit in exchange for their participation.

## Results

### Characterization of Sample

A community sample of 218 adolescents between 16 and 19 years of age consented to participation. They all filled in the SDQ and the SFI. A total of 143 out of 218 adolescents reported demographics, most likely the other ones did not do so as demographics were requested at the end of the survey after two unrelated questionnaires. As can be seen in Table 1, the educational background and age-range was diverse, with respect to gender, females were overrepresented. Therefore, it was vital we used age and gender specific norms for the SDQ subscales.

**Table 1:** Descriptive information on participants in Study 1 and 2

	Study 1		Study 2	
	N*	%	*N*	%
Gender:				
Female	100	70	85	72
Male	41	29	31	26
Not specified	2	1	2	2
Age:				
16	35	25	30	25
17	37	26	11	9
18	34	24	36	31
19	37	26	21	18
20	-	-	20	17
Educational level younger participants:				
Preparatory secondary vocational education	3	2	2	2
Senior general secondary education	15	11	0	0
University preparatory education	58	41	38	32
Educational level older participants:				
Intermediate vocational education	20	14	13	11
Higher Vocational Education	12	8	62	53
University preparatory education	-	-	2	2
University Education	35	25	1	1

* In Study 1, a total of 143 out of 218 adolescents reported demographics

As can be seen in Table 2, for all three SDQ subscales these age- and gender specific norms indicated that the “*normal*” category was significantly underrepresented. In addition, the “*abnormal*” category was significantly overrepresented, but only for emotional symptoms. A total of 41% instead of the expected 10% were in the “abnormal” category for emotional symptoms.

**Table 2:** Characterization of sample in terms of SDQ subscales. Column names contain expected percentages given gender and age specific norm tables

	“normal” (80%)	“borderline”(10%)	“abnormal”(10%)
Study 1			
Hyperactivity inattention	68%*	13%	19%
Conduct problems	73%*	15%	12%
Emotional symptoms	48%*	11%	41%*
Study 2			
Hyperactivity inattention	64%*	15%	20%*
Conduct problems	64%*	17%	19%
Emotional symptoms	42%*	20%	38%*

*: observed percentage deviates significantly (two-sided binomial test) from expected percentage given in column names

### Strategies

The inductive coding of the SFI yielded an inventory of 51 strategies (see Table 3). As also can be seen in Table 3, the deductive coding indicated that 15 of the 51 strategies were not, and 36 were, classified as instances of established emotion regulation or coping strategies. Notably a variety of 15 strategies e.g., *spending time with an animal*, *go for a stroll*, *spend time behind a screen* and *work-out*, were all categorized as the established strategy “distraction”.

**Table 3:** Strategies observed in Study 1, followed by additional strategies observed in Study 2. The other columns indicate whether a strategy could be coded as an established emotion regulation or coping strategy (*). If none of the strategies were coded as an established strategy, these were not included in the table (for example the strategy “humor” is not included)

Strategy	emotion regulation and coping strategies						
	problem solving	Emotional expression	emotional suppression	cognitive reappraisal	distraction	acceptance	avoidance
STUDY 1							
acknowledge or accept thoughts, feelings or emotions						*	
adjust cognition				*			
avoid certain foods or drinks							
avoid screen time							
await the situation							
be with an animal					*		
calm myself down			*				
count to ten							
distract myself					*		
do something creative or a hobby					*		
don’t express thoughts, feelings or emotions			*				
drink							
eat							
eliminate distractions	*						
express thoughts, feelings, emotions verbally		*					
find a solution	*						
get in touch with others		*					
get some fresh air					*		
go for a stroll					*		
ignore or avoid							*
isolate myself							*
just do the undesired behavior							
just don’t do the undesired behavior							
lie down or take a rest					*		
listen to music					*		
manipulate							
meditate or pray					*		
move around					*		
pay attention to breathing					*		
persevere							
physically release my emotions							
put thoughts, feelings or emotions aside				*			
reach out to other in a negative way							
read					*		
reward myself	*						
smoke					*		
spend time behind a screen					*		
start a conversation	*						
take a bath or shower					*		
take a break					*		
take a medicine							
think or reason	*						
think reassuring thoughts				*			
think stimulating or positive thoughts				*			
tidy or clean up					*		
try to get out of it							*
use a physical fix							
use an external fix							
walk away							*
workout							*
write down thoughts, feelings or emotions		*					
Totals study 1	5	3	2	4	16	1	5
STUDY 2 additional strategies							
make a plan	*						
do nothing about it							
use empathy							
find comfort							
make up/dress up					*		
change expectations				*			
ruminate							
fake it till you make it							
caffeine/energy drinks							
cannabis							
dance					*		
Totals study 2	1	0	0	1	2	0	0

Table 4 contains the two most often mentioned strategies per symptom, and their mean helpfulness ratings. Among these, only one strategy, *think or reason*, was reported in all three symptom domains. For hyperactivity/Inattention symptoms, the two most often mentioned strategies differed per symptom, although it was *Take a break* for 4 out of 5 symptoms. For conduct problems, the two most often mentioned strategies also differed between symptoms, although it was *start a conversation* for 3 out of 6 symptoms. Note that *manipulate* was also among the two often mentioned strategies, suggesting that participants reporting this strategy interpreted the question as “how do you do this” instead of “what do you do to manage this”. For emotional symptoms, the two most often mentioned strategies also differed between symptoms, although *get in touch with others* was mentioned for 3 out of 5 symptoms. In Table 4 it can also be seen that the mean helpfulness rating was 3, indicating to be regularly helpful, except for *physically release my emotions (*mean rating of 2).

**Table 4:** Per symptom the mean helpfulness of the two most often reported strategies in Study 1 (before /) and Study 2 (after /). For example, “Express thoughts, feelings, emotions” contains a score of 3/- for the worrying symptom, indicating that this strategy was in Study 1 among the two most often mentioned strategies for this symptom, and had a mean helpfulness of 3. Note if a second and third strategy were reported equally often, three strategies were included

	Hyperactivity/ Inattention^a^					Conduct problems^b^						Emotional symptoms^c^				
	restless	fidgeting	distracted	not thinking	focus	angry	disobedient	fight	lie or cheat	spoiled	steal	somatic	worrying	unhappy	nervous	scared /anxious
distract myself		-/3											- /3			
express thoughts, feelings, emotions													3/-			
find a solution			3/-													
get in touch with others													3/3	3/3	-/3	3/3
go for a stroll	3/3	-/3														
isolate myself						3/3										
just do the undesired behavior				3/3												
just don’t do the undesired behavior									-/3		3/4					
lie down or take a rest												3/3				
listen to music	-/3		-/3			3/3							-/3	3/3		
manipulate									3/-	3/-						
move around		3/-														
pay attention to breathing																-/3
persevere					3/3		-/3									
physically release my emotions								2/-		2/-						
start a conversation							3/-		3/-	3/-						
take a break	3/-	3/-	3/3		3/3											
take a medicine												3/3				
think or reason				3/3					3/3	-/3	3/3					3/-
think reassuring thoughts															3/-	
think stimulating or positive thoughts															3/3	
try to get out of it							3/3									
use a physical fix		3/-														
walk away								-/3								
workout								3/3		3/-						
use empathy										/3						

^a^: restless: “If I am restless or cannot stay still for long”; fidgeting: “If I am constantly fidgeting or squirming”; distracted: “If I am easily distracted, or if I find it difficult to concentrate”; not thinking: “ If want to do something without thinking”; focus: “If I realize that I cannot finish my work because I cannot maintain attention”

^b^: angry: “If I get angry or lose my temper”; disobedient: “If I do not want to do as I am told”; fight: “if I want to fight”; lie or cheat: “ If I want to lie or cheat; spoiled: “If I want to make other people do what I want”; steal: “if I want to take things that are not mine from home, school or elsewhere”

^c^: somatic: “If I have a headache, stomach-ache or sickness”; worrying: “If I worry”; unhappy: “If I am unhappy, depressed or tearful”; nervous: “ If I am nervous in new situations or if I lose confidence”; scared or anxious: “If I am scared or fearful”

## Interim Conclusion & Discussion

The purpose of Study 1 was to gather an understanding of adolescent- and symptom-specific strategies to manage psychopathology symptoms. First, inductive coding yielded a wide range of 51 strategies. Second, deductive coding indicated that a total of 36 out of 51 strategies could, and 15 could not, be categorized as established emotion regulation or coping strategies. Third, we showed that the two most often mentioned strategies differed between symptom domains and even between symptoms. Finally, we showed that for nearly^3^ all symptoms, the two most often reported strategies, were considered to be regularly helpful. This suggests that these strategies are deemed worthwhile by adolescents themselves.

In interpreting these findings, it is important to consider that less than expected adolescents in our sample score in the “normal” range on hyperactivity/inattention, conduct problems, and the emotional problems SDQ subscales. Especially emotional problems were more prevalent than expected. This may be explained by the fact that the study was performed during the COVID-19 lockdown which may have increased adolescents’ difficulties (Fitzpatrick et al., 2021; Hussong et al., 2021; Kuhlman et al., 2023; Pearcey et al., 2023; Scott et al., 2021). The lockdown may also have influenced the strategies adolescents were able to implement (Shanahan et al., 2023), for example joining parties or participating in organized sport activities was less feasible. For these two reasons - overrepresentation of problems and potential underrepresentation of some strategies – we performed Study 2.

## Study 2

Study 2 was performed to assess robustness of the results in Study 1 across different contexts, during and after the pandemic. In addition to the initial study’s objectives, Study 2 also aimed to study the relation between the goal adolescents intend to reach with a strategy and the helpfulness of a strategy. Specifically, we assessed whether adolescents implement strategies with the goal to solve a problem (problem-solving), avoid negative emotions (experiential avoidance) or avoid distressing thoughts (cognitive avoidance). Whereas several emotion regulation frameworks (Gratz & Roemer, 2004; Gross, 2015) point to the protective role of problem-solving strategies (Cuijpers et al., 2018; Michelson et al., 2022), the use of avoidance strategies has been related to increased psychopathology (Hussong et al., 2021; Rueda & Valls, 2017; Stone et al., 2019). Therefore, we aimed to investigate whether it is also true that self-reported problem-solving goals are positively, and self-reported avoidance goals are negatively, related to helpfulness.

## Methods

Methods were nearly equivalent to those in Study 1, exceptions are described below.

### Participants

We studied a community sample of adolescents aged 16 to 20 years.

### Materials

*Strengths and Difficulties Questionnaire (SDQ).* Reliability (Cronbach’s alpha) of the SDQ subscales was acceptable for hyperactivity/inattention (0.70) and emotional symptoms 0.67), whereas it was again low for conduct problems (0.44),

*Symptom-specific Fixes Instrument (SFI)* In the SFI we now also administered three goals items. That is, we incorporated the following questions: ‘With using this fix, was your goal to find a solution?’, ‘With using this fix, was your goal to avoid thinking about it?’, ‘With using this fix, was your goal to avoid your feelings?’. Participants could answer these questions with ‘yes’ or ‘no’ or could indicate that they did not fill out any fix.

### Procedure

Data were gathered post-pandemic. This period, and the six months preceding it, did not feature any COVID-19 limitations. In the survey, the questionnaires were administered in the following order: first demographics, then the SDQ, followed by the SFI, in which also the self-reported goals were assessed. On average it required 30 min to complete the whole survey. High school students filled in the survey in a classroom setting, a researcher monitored participants filled in the survey independently. University students filled in the survey in their own setting and were not monitored. To guarantee anonymity, we were not allowed to collect IP addresses.

### Data Analysis

#### Coding of Strategies

The data of 45% of participants was coded by three Dutch psychology students, two females and one male, aged 21 to 23 years. The remaining data of 55% of participants was coded by three Dutch psychology academics, all female, aged 33 to 59 years. To minimize disparities and disagreements, a calibration session was held using the data from a pilot survey, and data of 15% participants was double coded. Any disparities and disagreements were resolved by means of discussion. The coders coded responses into the 51 strategies obtained in Study 1. If this was not possible, they added additional categories to account for the possibility that additional strategies were used post-pandemic, it turned out that 11 additional strategies were required. We also assessed interrater reliability by means of Cohen’s Kappa (Cohen, 1960), separately for each of the 16 items of the SFI. To this end, one academic (female, 60 years of age) again coded, after 17 months, the answers of half of the participants (with uneven participant numbers) into the 62 potential codes. This coder also participated in a subset of the actual coding, but we reasoned that the 17-month delay may have resulted in forgetting.

#### Association Between Strategy Goal and Helpfulness

To investigate the hypothesis that the intended strategy goal predicts helpfulness, a linear multiple regression analysis was performed in SPSS. In the analysis, mean helpfulness was included as the dependent variable and the predictors were the mean problem-solving goal, avoidance of thinking goal and avoidance of feeling goal as predictors. To be able to report the evidence in support of the alternative hypothesis versus the null hypothesis, the Bayesian equivalent of a linear regression was performed in JASP (v0.19.3) using a uniform prior probability (JASP team, 2023).

## Results

### Characterization of Sample

The community sample consisted of 118 participants, between 16 and 20 years of age. They completed all questionnaires. As can be seen in Table 1, educational background was diverse, age was varied, and females were overrepresented. Therefore, it was vital we used age and gender specific norms for the SDQ subscales.

As can be seen in Table 2, for all three SDQ subscales these age- and gender specific norms indicated that the “*normal*” category was significantly underrepresented. In addition, the “*abnormal*” category was significantly overrepresented for hyperactivity/inattention symptoms and for emotional symptoms. For hyperactivity inattention, 20% instead of the expected 10% were in the “*abnormal*” category. For emotional symptoms this percentage was 38% instead of 10%.

### Strategies

In Table 3 it can be seen that, as compared to the first Study, 11 additional strategies were observed^4^. In Table 3 it can also be seen that 4 of these 11 strategies were, and 7 were not, classified as instances of established emotion regulation or coping strategies.

Table 4 contains the two most often mentioned strategies per symptom, note that only one of the 11 additional Study 2 strategies was among them. In Table 4 it can also be seen that only two strategies, *think or reason* and the strategy *listen to music* were reported in all three symptom domains.

For hyperactivity /inattention, the two most often mentioned strategies differed per symptom. For conduct problems, these also differed per symptom, although *think or reason* was mentioned for 3 out of 6 symptoms. For emotional symptoms, the two most often mentioned strategies also differed per symptom, although *get in touch with others* was mentioned for 3 out of 5 symptoms. In Table 4 it can also be seen that the mean helpfulness ratings were always 3 indicating that the strategy was considered to be “regularly helpful”. The only exception being *just don’t do the undesired behavior* to manage a conduct problems symptom, the mean rating of 4 indicating that this strategy was “often helpful”.

Table 5 gives inter-rater reliabilities. Averaged over the 15 SFI items, the interrater reliability was 0.71, which is good according to established criteria (Cicchetti, 1994). All SFI items had good to excellent reliabilities, except for three SFI items related to conduct problems, for which reliability was fair according to the same criteria.

**Table 5:** Interrater reliabilities for SFI items in Study 2

SFI item	Cohen’s Kappa
restless	.73^2^
fidgeting	.78^3^
distracted	.70^2^
not thinking	.70^2^
focus	.63^2^
angry	.77^3^
disobedient	.53^1^
fight	.74^2^
lie or cheat	.56^1^
spoiled	.59^1^
steal	.65^2^
somatic	.77^3^
worrying	.84^3^
unhappy	.88^3^
nervous	.73^2^
scared/ anxious	.75^3^
Mean over items	.71^2^

^0^: poor, ^1^: fair, ^2^: good, ^3^: excellent interrater reliability according to (Cicchetti, 1994)

### Association Between Strategy Goal and Helpfulness

The results of the linear multiple regression analysis showed that the model including the problem-solving goal, cognitive avoidance goal and experiential avoidance goal was not significant (F = 1.344, *p*=.260, R2=0.014). This indicates that none of the specific intended strategy goals predicted their helpfulness. The Bayesian equivalent of the linear multiple regression analysis confirmed these findings by showing that the data showed more support for the null model than for the model including the strategy goals, BF10 = 0.039. See Table the SOM, Table 1, for all Bayesian model comparisons.

### General Conclusion and Discussion

The purpose of the current studies was to gather an understanding of adolescent-specific strategies to manage mental health challenges. That is, we (1) generated an inventory of adolescent-specific strategies to manage psychopathology symptoms; (2) evaluated which strategies could, and which could not, be categorized as established emotion regulation or coping strategies; (3) evaluated whether strategies differed between symptoms, and (4) assessed whether adolescents considered their own strategies helpful. We did so in two studies in large community samples of adolescents aged 16 to 19 years from varying educational backgrounds. We conducted Study 1 during the pandemic and Study 2 post-pandemic. We conducted the second study to assess robustness of Study 1 results, as the pandemic-related lockdown may have increased mental health challenges and may have precluded strategies to manage them. In Study 2 we also assessed whether the goal of strategies was related to their usefulness.

With respect to our first aim, inductive coding in Study 1 yielded a wide range of 51 strategies, and in Study 2 we observed 11 additional strategies. Regarding our second aim, deductive coding indicated that in Study 1, a total of 36 out of 51 strategies matched established emotion regulation or coping strategies (Compas et al., 2017). In Study 2, a total of 4 out of the 11 additional strategies matched established strategies. These matching and non-matching strategies add to the literature in four ways. First, the matching strategies give detailed adolescent- and symptom-specific instances of established emotion regulation or coping strategies. For example, the emotion regulation or coping strategy “distraction” consists of strategies like *spending time with an animal*, *go for a stroll*, *spend time behind a screen*, and *work-out*. As these strategies are tailored to adolescents’ lives, they may provide useful input for prevention programs (Lansing et al., 2019). Second, and importantly, this diversity within established strategies suggests that it may be worthwhile *not* to lump these strategies into one established strategy, as one strategy may be more protective than another. As an example, the established strategy “distraction” contains both of *spending time behind a screen* and *work-out*, where the latter is more protective than the former (Hu et al., 2020; Ren et al., 2021; Rodriguez-Ayllon et al., 2019). Third, the non-matching strategies suggest that adolescents use an even richer repertoire of beneficial strategies to manage psychopathology symptoms than proposed in the emotion regulation and coping literature. For example, the reported strategy *avoid screen time* may indeed be beneficial (Eirich et al., 2022; Girela-Serrano et al., 2022). Finally, these results indicate that the Symptom-specific Fixes Instrument (SFI) should, as intended, be considered as an instrument to assess strategies adolescents use to handle symptoms of psychopathology, and not as an instrument to assesses coping or emotion regulation or strategies.

Concerning our third aim, we showed in both studies that hardly any of the two most often strategies cover all three symptom domains. This diversity in strategy use is in line with previous research showing that emotion regulation and coping strategies also differ between problem types (Compas et al., 2017; Zeman et al., 2002). The current studies add to this literature by showing that the two most often mentioned strategies even differ between symptoms of the same problem type. This suggests that it is useful to assess strategies in a problem-, and even symptom-specific way.

Finally, with respect to our fourth aim, we observed in both studies that for all symptoms, adolescents considered the two most often reported strategies, except for “Physically release my emotions,” to be helpful at least “regularly. This suggests that adolescents themselves deem these strategies worthwhile. This may be taken as first evidence that these strategies are protective. In Study 2, we also assessed whether helpfulness was related to the intended goals of the strategies, that is, problem-solving, cognitive avoidance, or experiential avoidance, which proved not to be the case. Based on emotion regulation frameworks (Gross, 2015), we expected that adolescents would report higher helpfulness for strategies with a problem-solving goal than for strategies with cognitive or experiential avoidance goals. The most obvious explanation for our null finding is that this association does not exist, or that the power to detect such associations was too low. However, there are two, not mutually exclusive, alternative explanations. The first one is that adolescents were not able to reflect on the goal of their strategy. The second alternative explanation is that adolescents evaluated helpfulness of their strategies both in the long-, as well as in the short-term. That is, the long-term effective problem-solving strategy may feel effortful and thus not helpful in the short-term, whereas the long-term less effective avoidance strategies may feel less effortful and thus more effective in the short term. (Aldao et al. 2010b; Cuijpers et al. 2018; Gratz and Roemer 2004; Gross 2015; Hayes and Hofmann 2021; Michelson et al. 2022; Suls and Fletcher 1985). Taking long- and short-term effectiveness together, adolescents then indeed would report no differences between the helpfulness of strategies with a problem solving or an avoidance goal. Future studies are needed to investigate this distinction between self-reported short- and long-term effectiveness.

Several potential limitations should be mentioned. First, in Study 1 students filled in the survey without supervision. As we did not collect IP addresses, we cannot rule out that students filled out the survey multiple times, or had someone else fill it in. Moreover, one may argue that some respondents were bots. We however deem this unlikely since the survey contained open ended questions, and was administered before public use of LLM’s, enabling responses to open ended questions, was possible (Protect Qualtrics Surveys from Bots - Research-IT). Fortunately, in Study 2 most respondents completed the survey under supervision. Because the results of the two studies are consistent, we deem it unlikely that lack of supervision compromised the Study 1 results. Second, note that in both studies, the reliability of the SDQ subscale on conduct problems was low, as has been reported before (Muris et al., 2003; Theunissen et al., 2019; Van Widenfelt et al., 2003). To our knowledge, there is no updated screener for conduct problems, although a recent study did provide guidance on how to enhance reliability by adapting SDQ subscales *(*Kankaanpää, 2023*).* Such an updated version may, if proven valid and reliable, be used in future studies. Note however that in the current studies, we only used the SDQ subscale score on conduct problems to characterize samples. Therefore, its low reliability did not affect the conclusions regarding our primary aims. Third, in Study 1, during the lockdown, less than expected adolescents scored in the “normal” range on hyperactivity/inattention, conduct problems, and the emotional problems SDQ subscales. The same pattern was observed in Study 2, after the lockdown. This underrepresentation of the “normal” range in both studies may be associated to the lockdown (Fitzpatrick et al., 2021; Pearcey et al., 2023; Racine et al., 2021; Scott et al., 2021) and its long-lasting effects (Kiviruusu et al., 2024). In addition, this underrepresentation may reflect self-selection, as participation in the studies was not mandatory (Keiding & Louis, 2018). Fourth, the inductive coding process allowed for responses to receive dual coding. This happened occasionally. For example, we labelled the response “I will go for a walk or workout” as both *going for a stroll* and *work out*. This single response thus contributed to the frequency count of two strategies. Fifth, in Study 1, the lockdown may have influenced the strategies adolescents were able to implement, however in Study 2, after the lockdown, we observed only 11 additional strategies, among which only one was among the two most often reported strategies. This large overlap suggests that strategies that were helpful in the lockdown were also helpful after it, and vice versa (Ashworth et al., 2022). Sixth, in both studies, the samples included a disproportionate number of females, which again may be due to self-selection. To account for this overrepresentation, we used gender specific norms for the SDQ. But for the strategies, we may have missed some male-specific strategies (Nolen-Hoeksema, 2012; Tamres et al., 2002), which may hinder generalization. Although this possibility can never be excluded, the fact that we managed to include 72 males in both studies combined, together with the observation that there were no marked gender-related differences in strategy use (see footnote 2), suggests that this possibility is not very likely. However, in future studies we recommend using a stratified sampling approach, for example by also including school tracks in which males are overrepresented. A final potential limitation is that, for some conduct problems symptoms, more than 50% percent of the participants reported never having experienced the symptom. Specifically, 69% in Study 1 and 70% in Study 2 reported never feeling angry; 89% in Study 1 and 84% in Study 2 reported never fighting or manipulating; 89% in Study 2 reported never lying, and 88% in Study 2 reported never stealing. Although adolescents received the instruction that they did not need to report a strategy, and many of them indeed did not do so for conduct problems symptoms, some adolescents may have felt compelled to do report a strategy, even if they did not experience the matching symptom. If all these adolescents also reported the same strategy, the two most often reported strategies may have been hypothetical. In future research it therefore may be worthwhile to only present an SFI question to participants that experienced the matching SDQ symptom.

Results from the current studies on adolescent- and symptom-specific strategies may provide input to develop in future studies a “strategies to manage psychopathology symptoms” questionnaire, in which adolescents rate how often they use each symptom-specific strategy. This questionnaire can then be used to quantitatively assess whether strategy use, either the type or the number, differs between adolescents with or without a psychopathology diagnosis. If this proves to be the case, it is relevant for four reasons. First, prevention programs can then strengthen strategies that typically developing adolescents predominantly use and reduce strategies that atypically developing adolescents report, that is, they can enhance the use of “protective strategies” (Eadeh et al., 2021; Moltrecht et al., 2021). These prevention programs can also be tailored to specific symptoms. For example, if typically developing adolescents more often use *get in touch with others* to manage symptoms associated with depression, prevention programs may include this strategy. In this manner, these programs are tailored to adolescents’ lives and needs, which is crucial for successful prevention (Aldao et al. 2010a; Freeman et al. 2015; Owen et al. 2012; Stilgoe et al. 2013). Second, the strategies questionnaire can be used to monitor whether protective strategies are used more often after prevention programs. Third, a personalized perspective (Sales et al., 2022; Weisz et al., 2011) can be taken in prevention by selecting from the protective strategies those that fit the individual best. For example, one adolescent may prefer the strategy *workout* while another may prefer *listening to music*. Fourth, the symptom-specific protective strategies may inform recent network-based prevention and intervention strategies, in which core symptoms, and not the entire condition, in a psychopathology network are treated (Mansueto et al., 2022).

Next to this future potential, one additional strength is that, to our knowledge, this is the first study on adolescent-and symptom-specific strategies in which we gathered answers to open-ended questions via an online survey. The varied nature of elicited responses - generating strategies which match established emotion regulation and coping strategies, as well as novel ways to manage symptoms - suggests that online elicitation of strategies is feasible. Although the coding of answers to open ended questions is still time-consuming, the collection of these data is not; for example, in the first study we gathered a dataset of over 200 participants within two weeks. Together this suggests that online elicitation may be a promising complementary approach, next to interviews and focus groups (Chang et al., 2023). Such an approach therefore can also be considered to study strategy use by children and adults, or even the development of strategy use from childhood to adulthood.

To conclude, in two studies we observed that adolescents employ a wide variety of strategies to manage mental health challenges. This can provide input for the development of a questionnaire assessing adolescent-and symptom-specific strategies to manage these challenges. Moreover, it may guide future preventative initiatives tailored to adolescents’ lives and needs.

## Supplementary Information

Below is the link to the electronic supplementary material.

Supplementary Material 1 (DOCX 237 KB)

## Data Availability

Not applicable, as qualitative data cannot be anonymized.

## Footnotes

1 Disorder-specific prevalence for 15–19 year olds: anxiety 5.3%, depression 3.4%, ADHD: 2.2%; conduct disorder: 1.8%% (World Health Organization, 2021).

2 Associated burden for anxiety and depression: educational problems, loneliness, suicide. Associated burden for ADHD and conduct disorders: educational problems, risk of criminal behavior (World Health Organization, 2021).

3 except for “Physically release my emotions”.

4 As a robustness check we tested whether there were gender differences in strategy use. That is, we tested for each of the 62 strategies whether the fraction of male participants using it differed from the fraction of males in the sample. Study 2 offered this opportunity, as all participants reported gender. The strategies “avoid certain foods or drinks” (p=.027), “just don’t do the undesired behavior” (p=.018) and ”cannabis” (*p*=.020) were used more often by males than females, whereas “express thoughts, feeling or emotions verbally” (*p*=.023) “get in touch with others” (p = < 0.001), “go for a stroll” (*p*<.001) and “make a plan” (*p*=.028) were used less often by males than females. If corrected for multiple testing, requiring a p-value < 0.05/62, only get in touch with others and go for a stroll differed between males and females.
