# Supplementary material for: Adolescents’ Strategies to Manage Mental Health Challenges In and After the Pandemic: Two Mixed-Methods Studies
Source: Res Child Adolesc Psychopathol. 2026 Jun 9;54(3):74. doi: 10.1007/s10802-026-01471-w (PMC13249706; doi:10.1007/s10802-026-01471-w)
Supplement: Supplementary file 1 — Supplementary Material 1 (DOCX 237 KB) [file 10802_2026_1471_MOESM1_ESM.docx]

SUPPLEMENTARY materials to:

adolescents’ strategies to MANAGE mental health challenges in and after the pandemic:

TWO mixed-methods studies

**COVID-regulations.**

In mid-July 2021 there was 1.5 meter distancing. In mid-August only 6 visitors were allowed. At the end of September the number of visitors was reduced to 3, the catering sector (bars, café’s, restaurants) closed at 10 PM and sport canteens closed. In mid-October the catering sector closed and no organized events were allowed. At the beginning of November people were advised to stay home, the number of visitors was reduced to 2, and all public spaces closed. In mid-December non-essential shops closed and there was only online education. From half of January 2022 onwards, including the data collection period, a lockdown was into effect: the catering sector, non-essential shops, entertainment venues, and indoor sports establishments closed; households were only allowed 1 visitor; outside homes it was allowed to gather with two people; there was a night-time curfew from 9 PM until 4.30 AM; and finally all schools only provided online education.

**Table S1:** *Bayesian linear regression model comparisons.*

| Models | P(M) | P(M\|data) | BFM | BF10 | R² |
| --- | --- | --- | --- | --- | --- |
| Null model | 0.250 | 0.761 | 9.554 | 1.000 | 0.000 |
| Problem-solving | 0.083 | 0.067 | 0.785 | 0.263 | 0.005 |
| Cognitive avoidance | 0.083 | 0.038 | 0.437 | 0.151 | 0.001 |
| Problem-solving + Cognitive Avoidance | 0.083 | 0.034 | 0.390 | 0.135 | 0.012 |
| Experiential avoidance | 0.083 | 0.033 | 0.370 | 0.128 | 0.000 |
| Problem-solving + Cognitive Avoidance + Experiential avoidance | 0.250 | 0.029 | 0.091 | 0.039 | 0.014 |
| Problem-solving + Experiential avoidance | 0.083 | 0.029 | 0.323 | 0.112 | 0.011 |
| Cognitive avoidance + Experiential avoidance | 0.083 | 0.009 | 0.104 | 0.037 | 0.003 |
